# The ORFeome of *Staphylococcus aureus *v 1.1

**DOI:** 10.1186/1471-2164-9-321

**Published:** 2008-07-07

**Authors:** Christina J Brandner, Richard H Maier, Daryl S Henderson, Helmut Hintner, Johann W Bauer, Kamil Önder

**Affiliations:** 1 Department of Cell Biology, University of Salzburg, Hellbrunner Strasse 34, A-5020 Salzburg, Austria; 2 Department of Pharmacological Sciences, School of Medicine, Stony Brook University, Stony Brook, New York, 11794, USA; 3 Division of Molecular Dermatology, Department of Dermatology, Paracelsus Private Medical University Salzburg, Salzburg, Austria

## Abstract

**Background:**

The bacterium *Staphylococcus aureus *causes significant morbidity and mortality in humans, primarily due to the emergence of strains that are resistant to antibiotics – notably methicillin-resistant *S. aureus *(MRSA) isolates. Development of effective strategies for the control and treatment of MRSA infections may best be achieved through 'omics' approaches, which first requires cloning the entire set of *S. aureus*' protein-encoding open reading frames (ORFs), or ORFeome.

**Results:**

The complete genome sequence of *S. aureus *strain Mu50 has 2697 predicted protein-coding ORFs. Based on the sequence of this strain we designed PCR primers to construct from an *S. aureus *(non-MRSA) clinical isolate an ORFeome library that contains 2562 unique Gateway^® ^entry clones (95% coverage), each corresponding to a defined ORF. The high quality of the ORFeome library was verified by DNA sequencing and PCR amplification, and its functionality was demonstrated by expressing recombinant proteins and observing protein interactions in a yeast 2-hybrid homodimerization screen.

**Conclusion:**

This first ORFeome library for *S. aureus *provides an essential new tool for investigating the systems biology of this important pathogen.

## Background

The number of completely sequenced bacterial genomes, including those of many pathogens, now stands at 640 (revised February 25, 2008) [1]. Such an extensive database provides not only the opportunity to define conserved open reading frames (ORFs) through a comparative genomics approach, but also whole-genome sequence information necessary to construct representative libraries of cloned ORFs, or 'ORFeomes', to enable large-scale, high-throughput 'omics' applications. In the case of hospital- and community-associated methicillin-resistant *Staphyloccocus aureus *(MRSA) strains, which are causing significant morbidity and mortality worldwide [2-7] the availability of an ORFeome should lead to an improved understanding of the molecular networks governing virulence, pathogenesis, and antibiotic resistance for the control and treatment of *S. aureus *infection [8-11].

The complete genome sequence of *S. aureus *strain Mu50 [12] encodes 2697 protein-coding ORFs (gi|47208328|dbj|BA000017.4| [47208328]). The massive undertaking to clone ORFs by the thousands cannot be easily achieved by conventional methods using restriction endonucleases. Therefore, we have used the Gateway^® ^site-specific recombinational cloning system [13] to construct an *S. aureus *ORFeome library of 2562 unique ORFs (95% coverage) in 'entry' vectors. Such entry vectors permit ORFs to be easily shuttled into other vector types; for example, into protein expression vectors to investigate protein-protein interactions by the yeast 2-hybrid (Y2H) assay [14]. We verified the quality of our *S. aureus *ORFeome library by PCR amplification and DNA sequencing. To demonstrate its functionality, we tested the production of His-tagged and GST-tagged recombinant proteins in *E. coli*. Additionally, we performed a Y2H analysis of 150 randomly chosen *S. aureus *ORFs to test for protein homodimerization, a property which is necessary for the correct functioning of many proteins.

Our repository of *S. aureus *ORFs offers a highly flexible platform with which to undertake high-throughput genomic and proteomic studies of *S. aureus *and MRSA infection, including the molecular mechanisms involved in transmission, virulence, immune-escape, and antibiotic resistance.

## Results

**Figure 1:**
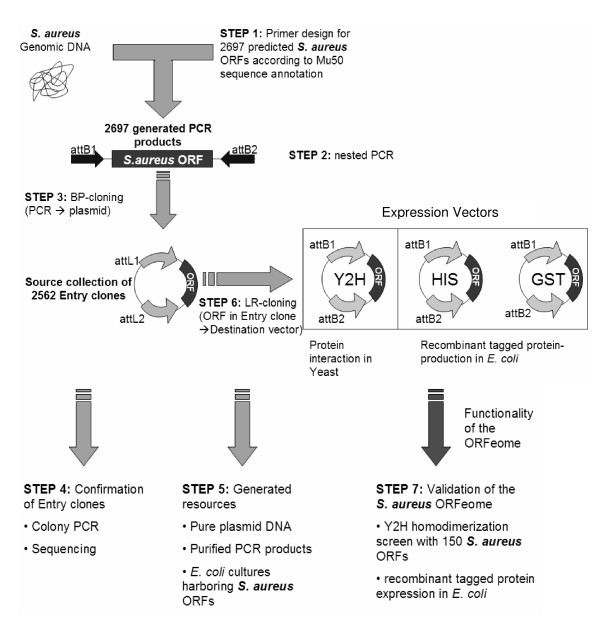
**Overview of the construction of the *S. aureus *ORFeome**. The PCR products of the amplified ORFs were used in the first step for a BP reaction, followed by PCR and sequencing for technical confirmation. Finally, generation of a library and validation by functional analysis were carried out.

The overall strategy we have employed for construction and characterization of the ORFeome of *S. aureus *is summarized in Figure 1 [see also Additional file 1].

### Primer design

In addition to a 20- to 30-nucleotide-long ORF-specific sequence, each PCR primer was designed to generate a Gateway^®^-compatible attB1 (forward primer) or attB2 (reverse primer) recombination site [13,15] flanking the amplified ORF [see Additional file 2]. Full-length attB sites at the 5' and 3'ends were generated using secondary universal adapter primers in the same PCR. A one-step adapter PCR method was used, instead of the common two-step adapter PCR procedure, to reduce the required cloning steps [see Additional file 2]. To allow for the production of C-terminal fusion proteins, which can be expressed from appropriate destination vectors, the stop codons of each ORF were omitted in the reverse primers. Shine-Dalgarno (5'-GAAGGAGATA-3') and Kozak (5'-ACCATG-3') consensus sequences were incorporated into the forward primers such that the ATG in the Kozak sequence is in frame with the attB1 site, thus allowing N-terminal fusion proteins to be produced from destination vectors that contain N-terminal tags.

### Gateway^® ^recombinational cloning

The PCR products were inserted into the Gateway^®^-compatible vector pDONR™/Zeo (Invitrogen) by BP-cloning. The products resulting from site-specific recombination were transformed into *E. coli*. A portion of the cells was plated on solid medium containing Zeocin™, and the remainder was used to inoculate liquid medium containing Zeocin™ to generate bacterial stocks of pooled entry clones for long-term freezer storage. On solid medium, ~90% of the plates yielded at least 50 colonies; lower numbers were obtained with inserts >3 kb. A single colony from each plate was tested in a colony-PCR with pDONR™/Zeo-specific primers. The resulting PCR products were detected by gel electrophoresis and ethidium bromide staining. Transformants which had an insert of the expected size were picked and grown in liquid medium containing Zeocin™ to generate bacterial glycerol stocks for long-term freezer storage. By this approach we obtained 1753 transformants carrying the correct insert, which represents 65% of the 2697 predicted ORFs. Using both the positive single-colony glycerol stocks to inoculate liquid medium containing Zeocin™ and the liquid glycerol stocks for those ORFs for which no positive single-colony isolate was obtained, cultures were grown in 96-well plates and plasmid DNAs were prepared and used for PCR. For 2562 (95%) samples, an insert of the size expected for the ORF was observed. The remaining 5% yielded a PCR product of ~300 bp, derived from empty pDONR™/Zeo vector. In light of this result, we expect that additional positive single transformants may be present in the liquid glycerol stocks. The single-colony glycerol stocks, the glycerol stocks of the pooled transformants, and the purified plasmid clones together constitute the ORFeome of *S. aureus*.

### Validation of entry clones by DNA sequencing

To verify that the inserted ORFs are in the correct reading frame and indeed correspond to the assigned identity, we took a random sample of 300 plasmid preparations and subjected them to sequencing with a pDONR™/Zeo-specific forward primer. All 300 samples were found to be correctly inserted, and a BLAST search of each sequence against the *S. aureus *Mu50 genome (NCBI Refseq: NC_002758) confirmed the identity of each ORF.

### Validation of entry clones by recombinant protein production

**Figure 2:**
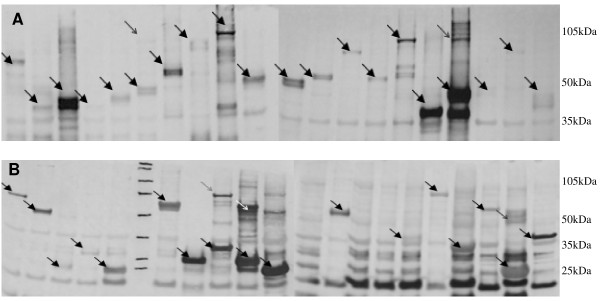
**Recombinant protein expression in *E. coli***. (A) Western blot of 20 GST-tagged *S. aureus *recombinant proteins detected with anti-GST antibodies. (B) 17 of 20 recombinant His-tagged *S. aureus *proteins could be detected with anti-His antibodies on a Western blot. Equal amounts (2.5 μg) of total protein from crude lysates were loaded in each lane. The arrows indicate the recombinant proteins.

Another random sample was used to show expression of proteins from the cloned ORFs. Forty different entry clones were each subjected to a recombination reaction, the LR-Reaction, which recombines the attL-sites on the entry clones with the attR-sites on the destination vectors [15]. Twenty of the ORFs were shuttled into the pDEST™15 vector (Invitrogen) designed to make a fusion protein with a GST-Tag, and the other 20 entry clones were used in an LR-Reaction with the pDEST™17 vector (Invitrogen), creating fusion proteins with a 6 × His-Tag. The resulting products were transformed into the *BL21*(DE3) protein expression strain of *E. coli*. After induction of protein expression with IPTG, the cells were lysed and 2.5 μg total protein from the crude lysates were analyzed by Western blot and antibody detection with biotin-linked anti-His and anti-GST antibodies. Protein was expressed from all 20 of the GST-tagged proteins and 17 of the 20 His-tagged proteins (Figure 2).

### Yeast two-hybrid homodimerization screen

**Figure 3:**
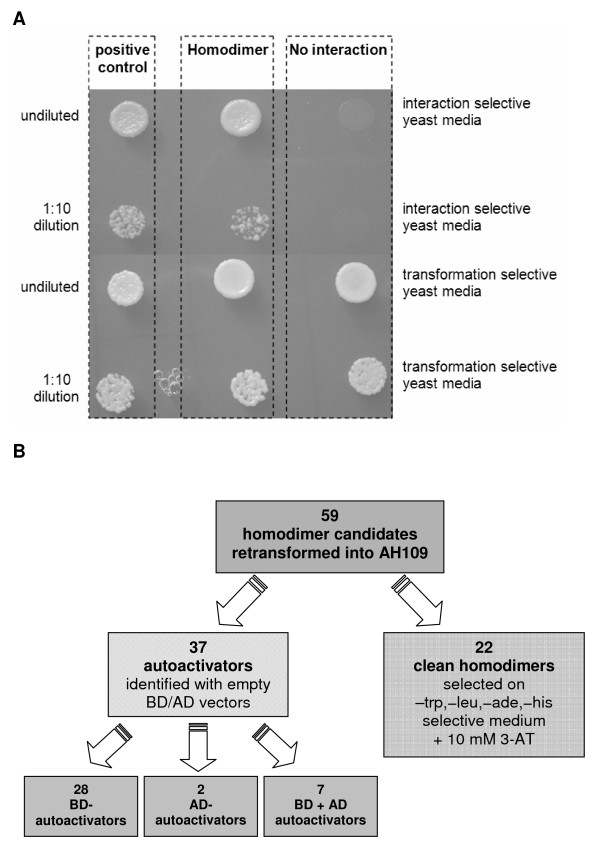
**Y2H screen for homodimers**. (A) Yeast cells harboring bait and prey plasmids were resuspended in sterile water and dropped onto protein interaction selective yeast medium. The positive control shows the interaction of the p53 and SV40-large T-antigen (Y2H internal controls) and 1 of the 22 found homodimers in this study. A negative interaction obtained in the screening is also shown. To test for the presence of both plasmids, the yeast cells were also plated on plasmid-selective medium lacking tryptophan and leucine. Y2H positive colonies can grow on synthetic complete yeast media lacking histidine, adenine, tryptophan and leucine. (B) Flowchart showing the results of retesting candidate homodimers under stringent selection conditions. Fifty-nine candidate homodimers were retransformed into AH109 as follows: i) AD-ORFs + DB empty vector; ii) DB-ORFs + empty AD vector; and iii) DB-ORFs + AD-ORFs. The transformations were plated on nutritionally selective medium deficient for tryptophan, leucine, histidine, and adenine, plus 10 mM 3-AT. All 22 homodimers were confirmed as "clean homodimers." The numbers of each type of autoactivator are indicated.

Numerous proteins must undergo homodimerization before they can carry out their functions. For example, many receptors in metabolic pathways are homodimers or form homodimers after ligand binding. In fact, screening for new ligands or ligand-competing drugs is sometimes performed by assaying for homodimerization [16]. As a functional test of our *S. aureus *ORFeome library, we shuttled 150 entry clones into Gateway^®^-compatible Y2H destination vectors to screen for homodimerization in yeast. After transforming AH109 (Mat a) cells with DNA binding domain (DB) vectors (pBD-Gate2) and Y187 (Mat α) cells with activation domain (AD) vectors (pAD-Gate2), colonies growing on each plate were mated on YPD plates. Yeast colonies appeared on 59 plates deficient in the reporter supplements adenine and histidine, and in the vector-encoded amino acids tryptophan and leucine, indicating 59 putative homodimers (Figure 3). However, the Y2H system is prone to autoactivation artifacts, in which a protein expressed from a single vector, rather than as protein-protein dimer from complementary bait and prey vectors, is able to reconstitute a functional transcription factor to permit growth of the yeast. Therefore, tests for autoactivation were done to identify such false positives. For this test, AH109 yeast cells transformed with DB vectors were plated on nutritionally selective medium deficient in tryptophan, adenine, and histidine. Y187 yeast cells pre-transformed with AD vectors were mated with yeast AH109 transformants containing an empty DB vector and plated on nutritionally selective medium deficient in tryptophan, leucine, adenine, and histidine. Thirty-seven of the original 59 putative homodimers failed this test (i.e., colonies were observed); moreover, the same results were obtained in the added presence of 10 mM 3-amino-1,2,3-triazole (3-AT), a more stringent growth condition (Figure 3B). We consider the remaining 22 newly discovered homodimerizations to be genuine protein-protein interactions in *S. aureus *(Table 1). To obtain support for this assumption, we searched the literature for reports of dimerization involving homologs of these 22 proteins in other species. We found that at least 6 of the 22 proteins have highly conserved dimer-forming homologs (Table 1), based on biochemical and structural data.

**Table 1:** Twenty-two newly described *S. aureus *homodimers listed by protein size.

Clone	Protein name	size	Interolog
SAV2407	**Putative uncharacterized protein**	88 AA	
SAV1893	**Ferritin**	166 AA	
SAV2249	**50S ribosomal protein L4**	207 AA	
SAV1233	**Ribonuclease 3**	243 AA	[33]
SAV2701	**Similar to vraD protein**	252 AA	
SAV0647	**Ferrichrome transport ATP-binding protein**	265 AA	
SAV2247	**50S ribosomal protein L2**	277 AA	
SAV1330	**Homoserine kinase**	304 AA	[38]
SAV0217	**Similar to NADH-dependent dehydrogenase**	359 AA	
SAV1737	**Chorismate mutase homolog**	363 AA	
SAV1235	**Signal recognition particle**	416 AA	
SAV1627	**Similar to ATPase, AAA family**	424 AA	
SAV1454	**Asparaginyl-tRNA synthetase**	430 AA	[39]
SAV2121	**Transcription termination factor Rho**	438 AA	[40]
SAV1576	**Putative 2-methylthioadenine synthetase**	448 AA	
SAV1900	**Glutamyl-tRNA(Gln) amidotransferase subunit A**	485 AA	
SAV2081	**Probable DEAD-box ATP-dependent RNA helicase**	506 AA	
SAV0205	**Oligopeptide transport ATP-binding protein**	530 AA	
SAV2029	**60 kDa chaperonin**	538 AA	[41]
SAV0700	**Fructose specific permease**	652 AA	
SAV2157	**Putative uncharacterized protein**	710 AA	
SAV1905	**ATP-dependent DNA helicase pcrA**	730 AA	[42]

## Discussion

The ORFeome of *S. aureus *that we constructed in this work consists of 1753 ORFs maintained as homogeneous plasmid DNAs purified from single colonies plus 809 ORFs purified from separate 'pools' of entry clones. These 2562 cloned ORFs represent 95% of the *S. aureus *(Mu50) ORFeome, a proportion of coverage similar to those reported for the ORFeomes of three other bacterial pathogens, *Brucella melitensis *[17], *Treponema pallidum *[18], and *Francisella tularensis *[19]. PCR amplification of the ORFs produced products not only of the expected size, but also shorter ones, which were probably primer dimers [20]. Although we did not purify the PCR products as in other ORFeome projects [21], enough material was produced for individual BP reactions, all of which resulted in colonies after transformation. Testing of the effectiveness of both purified and unpurified PCR products in BP reactions showed that cloning could be achieved to the same extent with both types of PCR products. To minimize the occurrence of PCR-induced mutations, we optimized the PCR conditions, used a proofreading-capable DNA polymerase, and kept the number of amplification cycles low.

Entry clones are the basis for further experiments using recombinational cloning, including genome-wide protein-protein interaction (PPI) analysis, protein expression, and protein localization studies [22-26]. The single entry clones are of higher quality than the pools of entry clones because they are homogeneous, and LR reactions performed subsequently with the pure clones will be more efficient. However, for reasons of economy and because of time constraints, it was not possible to screen every colony to obtain a pure isolate of the appropriate ORF-containing entry clone. When the first colony-PCR failed to produce the anticipated ORF, we screened the remaining pool of colonies to identify the presence of the expected entry clone. We failed to produce PCR products for 135 ORFs. This could be due to possible errors in annotation of the Mu50 genome or, more likely, because of nucleotide differences between our clinical *S. aureus *isolate and the sequenced Mu50 genome or for technical reasons related to primer design and effectiveness specific to each missing ORF.

Different strains of *S. aureus *can exhibit important differences in gene content owing to the presence/absence of plasmids and/or mobile elements integrated into the chromosome [e.g., staphylococcal cassette chromosome (SSC) elements, prophage], usually at specific loci termed "genomic islands" [27]. Such factors are important determinants of MRSA virulence and drug resistance, so that an ideal ORFeome would include ORFs for these genes in addition to the bacterial core ORFs. Our ORFeome is certainly incomplete in this regard, because it is a composite of only two *S. aureus *strains; however, ORFs for virulence and resistance factors of interest could easily be added to the ORFeome as the resource is further characterized and developed.

We assessed the quality of the ORFeome by PCR and agarose gel-electrophoresis of the PCR-products for all the entry clones obtained. Sequencing a sample of 300 PCR products confirmed the identity of the entry clones. Functional validation was done by recombinant protein production and PPI analysis using the Y2H system. In addition to identifying 22 new homodimeric interactions, the experiments also showed the functionality of the ORFeome and its suitability for further applications, including automated high-throughput protein purification or ORFeome-wide PPI analysis. Indeed, generation of an *S. aureus *intrapathogen 'interactome' is conceivable through ORFeome-wide PPI screening of 3.3 million (2562 × 2562/2) protein-protein interactions by automated procedures. Likewise, a system-wide screen for host-pathogen PPIs could be conducted by screening against other available ORFeomes, e.g. the human ORFeome [28], which would produce a detailed description of the host-pathogen interface at the molecular level, leading to improved understanding of *S. aureus *pathogenesis in humans.

Production of recombinant proteins in the present system is achieved with the two well-known protein fusion tags, 6 × His and GST. These tags could be used for further protein purification or affinity studies such as GST Pull-Down [29] for validation of protein-protein interactions. We successfully produced fusion proteins with both types of tags, although the GST-tagged proteins were more strongly expressed on average than the His-tagged ones in our experiments. His-tagged proteins could not be detected in three out of 20 experiments by Western blotting, due to high background staining caused by the His-specific antibody used or because of low expression rates, or both. Although, we loaded equal amounts of total *E. coli *protein lysates on protein gels, the intensity of the expressed proteins varied because of degradation in the crude cell lysates. It is interesting to note the presence in some experiments of a second band double the size of the expected molecular weight, suggesting homodimerization, although it is unlikely that dimerization would occur under the denaturation conditions used.

We tested 150 proteins for homodimerization using the Y2H-system and found a total of 22 such interactions (Table 1) after ruling out 37 autoactivators. All Y2H-experiments, including the autoactivator tests, were performed in triplicate, and each PPI was reproducible by independent analysis. It is important to stress that even in the cases of autoactivation, an ORF had to have been expressed in the yeast reporter strain, which again demonstrates the functionality of the ORFeome. The 22 homodimers observed here imply that at least ~15% (22 of 150) of the tested proteins can undergo homodimerization, consistent with the levels found in previous large-scale studies (2–20%) of different organisms [*S. cerevisiae *3.6%, *C. elegans *2.8%, *D. melanogaster *2.2%, and *H. sapiens *19.6% [30]; 143 homodimers among 1546 PPIs (9.25%, Y2H-dataset) in *H. sapiens *[31]; 33 homodimers in 1301 human PPIs (2.5%) [32]. Moreover, in the literature we found reports of biochemical and/or structural evidence for homodimerization among homologs (Interologs) of 6 of our 22 dimer-forming proteins (Table 1). For example, RNase III, the product of the SAV1233 clone, is a homodimer in numerous species [33]. In *S. aureus*, RNase III is an essential regulator of expression of the *spa *gene, which encodes the virulence factor Staphylococcal protein A[34].

## Conclusion

The cloning of 95% of the *S. aureus *ORFeome was achieved, and a random sampling of ORF-containing plasmid vectors showed the ORFs to be functional in two different downstream applications: recombinant protein production in *E. coli *and a protein interaction assay (Y2H). The availability of the *S. aureus *ORFeome with appropriate Gateway^®^-compatible destination vectors offers many possibilities for proteome-wide studies of such medically important phenomena as virulence and antibiotic resistance in a high-throughput manner. This first version of the *S. aureus *ORFeome can be extended in future work by addition of the missing ORFs.

## Methods

### Primer Design

The DNA sequence of *S. aureus *strain Mu50 was obtained from the NCBI Genome Database (NCBI; Refseq: NC_002758). Primary gene-specific forward primers were designed by adding the sequence 5'-AAAAAGCAGGCTTGGAAGGAGATAGAACCATG-3' to the 5' end of the first 20 to 30 nucleotides of each ORF. Gene-specific reverse primers were constructed by adding the nucleotides 5'-GTACAAGAAAGCTGGGTA-3' to the 5' end of the last 20 to 30 nucleotides of the complementary strand of the ORF. The gene-specific parts of the primary forward and reverse primers were chosen to give a similar annealing temperature during the PCR, between 40–60°C. The 2697 primer pairs were obtained from Illumina Inc. in a 96-well format.

### Isolation and preparation of *S. aureus *genomic DNA

Genomic DNA of *S. aureus *was prepared from a fresh overnight culture of a clinical isolate of *S. aureus *(non-MRSA; the strain is sensitive to ampicillin) using the GenElute™ Bacterial Genomic DNA Kit (Sigma-Aldrich). This isolate was originally identified as *S. aureus *by standard microbiological procedures in a hospital microbiology laboratory (University Hospital Salzburg). After cell harvesting, the pellet was resuspended in resuspension solution containing 3125 U/μl lysozyme and 62.5 U/ml lysostaphin and further prepared and purified according to the product protocol manual. The resulting genomic DNA had a concentration of 200 ng/μl and a 260/280 absorbance ratio of 1.85.

### PCR amplification of the ORFs

Each of the 2697 PCRs were performed in 96-well plates containing 50-μl assay volumes consisting of 1.25 U Platinum Pfx polymerase (Invitrogen), 1 mM MgSO_4_, dNTP mix (0.3 mM each), primary forward and reverse primers (0.1 μM each), secondary adapter forward and reverse primers (0.4 μM each), 10× Pfx amplification buffer (5 μl) and *S. aureus *genomic DNA (200 ng). The sequence of the secondary forward adapter primer is 5'-GGGGACAAGTTTGTACAAAAAAGCAGGCTTG-3' and that of the secondary reverse adapter primer is 5'-GGGGACCACTTTGTACAAGAAAGCTGGGTA-3'. After the first few cycles, enough DNA template was produced by the primary forward and reverse primers to enable the secondary adapter primers to bind and extend the first PCR products to generate the full-length attB1 and attB2 sites flanking the ORFs. All 4 primers were used together in the same PCR. The 25 PCR cycles (94°C for 30 s, 48°C for 30 s, and 72°C for 1 min/kb) were preceded by heating to 94°C for 5 min and were followed by a 7-min incubation at 72°C. PCR products were used immediately without purification and then stored at -20°C.

### attB × attP recombination reactions – BP reactions

The Gateway^®^-compatible amplified ORFs were recombined into the vector pDONR™/Zeo (Invitrogen) by using the BP Clonase™ II Enzyme Mix (Invitrogen). In 96-well plates, samples containing 2 μl unpurified PCR product, 1 μl BP Clonase™ II Enzyme Mix, 150 ng pDONR™/Zeo plasmid and TE buffer, pH 8.0, up to 10 μl were incubated overnight at 25°C. After adding 1 μg proteinase K (Invitrogen) and incubating at 37°C for 30 minutes, the BP reactions were directly used for bacterial transformation. The reactions were stored at -20°C.

### Transformation

A 3 μl aliquot from each of the 2697 BP reactions was added to One Shot^® ^TOP10 chemically competent *E. coli *using the manufacturer's protocol and 96-well plates. After heat-shock at 42°C for 30 s, 50 μl of SOC medium (Invitrogen) was added to the transformation reactions and the samples were incubated 1 h at 37°C. After the incubation, 20 μl of each sample were plated onto low-salt LB solid medium containing 80 μg/ml Zeocin™ (Invitrogen) and incubated overnight at 37°C to produce single colonies. The remainder of the transformation reaction was used to inoculate 150 μl of low-salt LB liquid medium containing 80 μg/ml Zeocin™. These cultures were also incubated at 37°C overnight to generate transformants for long-term storage by adding glycerol and freezing at -80°C.

### Colony PCR of bacterial clones

A single colony from each transformation reaction was analyzed by PCR to verify the correct size of the inserted ORF. The 2697 PCRs were performed in 96-well plates containing 50-μl samples with 2.5 U BioThermRed™ Polymerase (Genxpress), pDONR™/Zeo-specific forward primer (5'-GTAAAACGACGGCCAG-3') and reverse primer (5'-CAGGAAACAGCTATGAC-3') (0.3 μM each), dNTP mix (0.2 mM each) and 10× BioTherm™ reaction buffer (5 μl). Colonies were picked with a sterile pipet tip and transferred to the wells of the 96-well plate. The 45 PCR cycles (94°C for 30 s, 55°C for 30 s, and 72°C for 1 min/kb) were preceded by heating to 94°C for 5 min and followed by a 7-min incubation at 72°C. The sizes of the PCR products were determined by agarose gel electrophoresis and ethidium bromide staining. Entry clones with inserts having the expected size were inoculated in 150 μl low-salt LB liquid medium containing 80 μg/ml Zeocin™ and incubated overnight at 37°C to generate glycerol long-term frozen stocks. In cases where the PCR failed to verify a positive colony, 'pooled' entry clones were analyzed. An aliquot of the entire transformation reaction was cultured in liquid LB medium overnight at 37°C. The entire overnight culture was subject to plasmid isolation and tested again by PCR. Plasmid preparations from cultures that were positive by PCR indicated the presence of correctly cloned ORFs; at worst, they consisted of a mixed population of cells having empty plasmids and ORF-containing plasmids.

### Glycerol long-term frozen stocks and plasmid preparation

Overnight cultures were mixed with glycerol to a final glycerol concentration of 40% for long-term storage at -80°C. One millilitre of low-salt LB liquid medium containing 80 μg/ml Zeocin™ was transferred into 2-ml deep-well plates (Nunc) and inoculated with 2 μl of overnight bacterial culture. After overnight growth at 37°C, plasmids were purified with a ChargeSwitch^® ^NoSpin Plasmid Micro Kit (Invitrogen) in a 96-well plate format.

### DNA sequencing

Sequencing of plasmid DNAs was performed on 300 randomly chosen PCR-positive ORFs. Standard dideoxy sequencing was used [35,36]. A 10-μl sample containing 5 μl plasmid preparation, the same pDONR™/Zeo forward primer as above (0.32 μM), 2 μl BigDye^® ^Terminator v3.1 Ready Reaction Mix (Applied Biosystems) and 1 μl 5× sequencing buffer was heated to 95°C for 5 min followed by 25 cycles of extension reactions (95°C for 10 s, 50°C for 5 s, and 60°C for 90 s). After precipitating with sodium acetate and absolute ethanol, the redissolved DNA was sequenced with an ABI 3130 Genetic Analyzer Capillary Array (Applied Biosystems).

### attL × attR recombination reactions – LR reactions

Entry vectors were set up in LR reactions to recombine the gene of interest into several destination vectors. The destination vectors used were pDEST™15, pDEST™17 (both from Invitrogen), pBD-Gate 2, and pAD-Gate 2 [37]. The two Gate vectors are in-house-generated Gateway^®^-compatible pGADT7 and pGBKT7 plasmids of the Matchmaker™ GAL4 Two-Hybrid system (Clontech) for yeast two-hybrid analysis. Samples containing 5 μl prepared entry clone, 1 μl LR Clonase™ II Enzyme Mix (Invitrogen), destination vector (150 ng), and TE buffer, pH 8.0, to 10 μl were incubated at 25°C for two hours. After adding 1 μg proteinase K (Invitrogen) and incubating at 37°C for 30 min, the LR reactions were directly used for plasmid transformation into *E. coli*. The reactions were stored at -20°C. Bacteria were transformed with the LR reactions as described above using the appropriate antibiotic: 100 μg/ml ampicillin for pDEST™15, 17, and pAD-Gate2; and 50 μg/ml kanamycin for pBD-Gate2. Colony PCRs were performed as described above to verify the successful cloning by using vector-specific primers. The expression clones were prepared using a Plasmid Purification NoSpin Plasmid Micro Kit (Invitrogen).

### Recombinant protein production

Prepared pDEST™15 and 17 expression vectors, which harbor the desired ORFs, were transformed into chemically competent *E. coli *BL21(DE3) (Invitrogen) and plated onto LB solid medium containing 100 μg/ml ampicillin. Single colonies from each plate were used to inoculate 3 ml LB liquid medium containing 100 μg/ml ampicillin. Cells were grown at 37°C until the OD_600 _reached 0.4–0.6, and protein expression was induced by adding IPTG (0.5 mM) and incubating for a further two hours. The cells were lysed and protein expression was shown by SDS-PAGE and Western blot analysis with antibody detection.

### Western blot analysis

Proteins were denatured at 95°C for 5 min and separated on a gel (NuPAGE^® ^10% Bis-Tris Gels (Invitrogen)) at 165 V for 90 min. Buffers used were from Invitrogen (NuPAGE^® ^SDS running buffer, and LDS sample buffer). Proteins were electroblotted onto a Hybond-ECL™ nitrocellulose membrane (Amersham Biosciences) with blotting buffer (3 g Tris, 14.4 g glycine, 200 ml methanol, 10 ml 10% SDS in 1000 ml water, pH 8.4) at 60 V for 1 h.

Membranes were blocked with 5% BSA (Calbiochem, San Diego, CA) in TBS + 0.3% Tween-20^® ^(Sigma-Aldrich) pH 7.4, at room temperature for 1 h, followed by incubation with first antibodies for 1 h. Two 5-min washing steps with TBS were followed by incubation with secondary antibodies for 1 h. After four final washing steps with TBS (10 min each), Western-blue stabilized substrate alkaline phosphatase (Promega) was added. Primary antibodies: Mouse anti-GST (B-14): sc-138 B and mouse anti-His His-probe (H-3): sc-8036 B (Santa Cruz Biotechnology, Santa Cruz, CA); both antibodies are biotin conjugates. Secondary antibodies: anti-mouse IgG – alkaline phosphatase-conjugated (Sigma-Aldrich).

### Yeast 2-hybrid analysis – yeast transformation and mating

Prepared expression vectors containing the desired ORFs were transformed into the yeast strains AH109 (Mat a) and Y187 (Mat α) (both Clontech) and first plated on a nutritionally selective plate deficient in tryptophan for pBD-Gate2 transformants and deficient in leucine for pAD-Gate2 transformants. One hundred and fifty baits (pBD-Gate2 Y2H expression vectors harboring the bacterial ORFs) were transformed into AH109 (Mat a) and 150 preys (pAD-Gate2 Y2H expression vectors harboring the bacterial ORFs) were transformed into Y187. After incubation for 5 days at 28°C, up to 5 colonies from each transformation reaction were used to inoculate the corresponding liquid medium and incubated at 28°C until the cultures reached turbidity. Additionally, glycerol stocks were prepared for long-term storage by adding an equal volume of 100% glycerol to the liquid cultures. For the mating procedure, 5 μl of liquid cultures of bait and prey of opposite mating type were brought together on a YPD plate and incubated overnight at 28°C. The next day a small amount of cell material was dissolved in 100 μl of autoclaved distilled water and one-half of each was plated onto a nutritionally selective plate deficient in tryptophan and leucine to test for positive mating and on a nutritionally selective plate deficient in tryptophan, leucine, adenine, and histidine to test for the putative interaction.

## Authors' contributions

KÖ provided the original concept of the study, supervised the study and contributed to writing the paper. CJB and RHM performed all working steps, were responsible for the data analysis and the manuscript preparation. DSH provided comment and intensive revisions to the manuscript. JWB and HH gave scientific support to the manuscript. All authors read and approved the manuscript.

## Supplementary Material

Additional file 1 **ORFeome Flowchart**. A detailed flowchart describing the whole process: PCR, BP reactions, transformation, single colonies or minipools, glycerol stocks, colony-PCR and plasmid PCR, and sequencing verification of all the clones. Click here for file

Additional file 2 **Gateway recombination site attachement**. This file shows the scheme of the one step adapter-PCR to create Gateway^® ^adaptable attB PCR products. Click here for file
