# Supplementary figures and images for: The ORFeome of Staphylococcus aureus v 1.1
Source: BMC Genomics. 2008 Jul 7;9:321. doi: 10.1186/1471-2164-9-321 (PMC2474624; doi:10.1186/1471-2164-9-321)

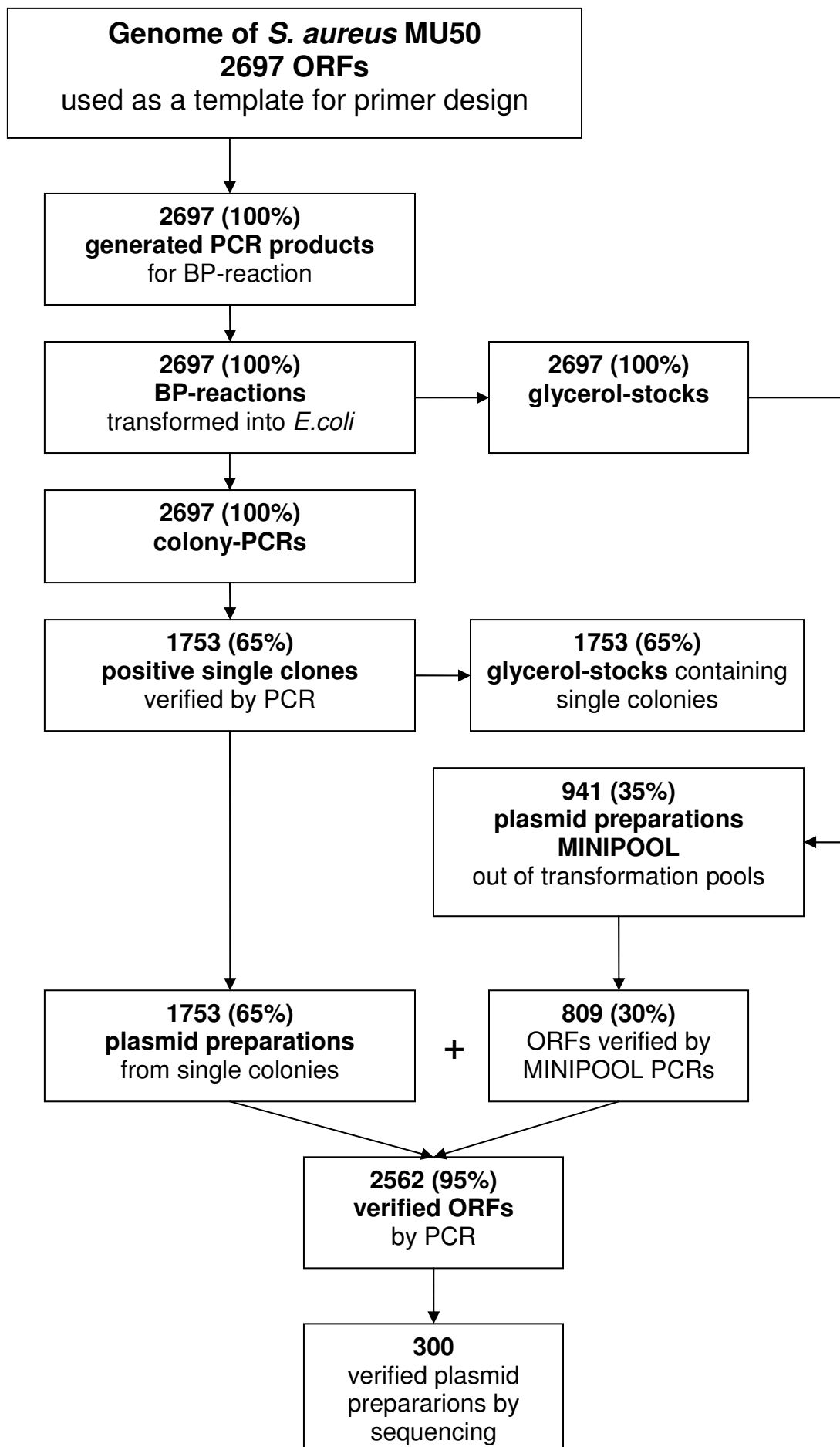

Supplement: Additional file 1 — ORFeome Flowchart. A detailed flowchart describing the whole process: PCR, BP reactions, transformation, single colonies or minipools, glycerol stocks, colony-PCR and plasmid PCR, and sequencing verification of all the clones. [file 1471-2164-9-321-S1.pdf]

**A**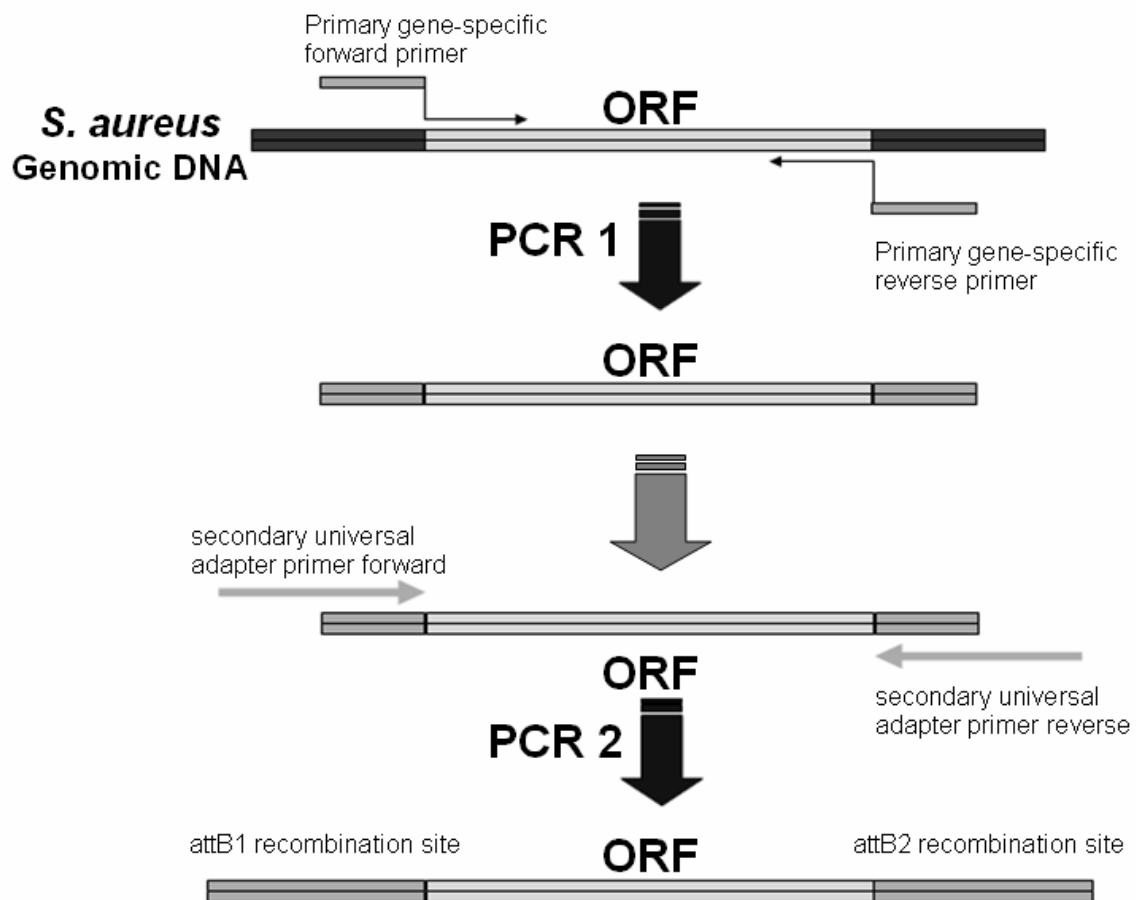**B**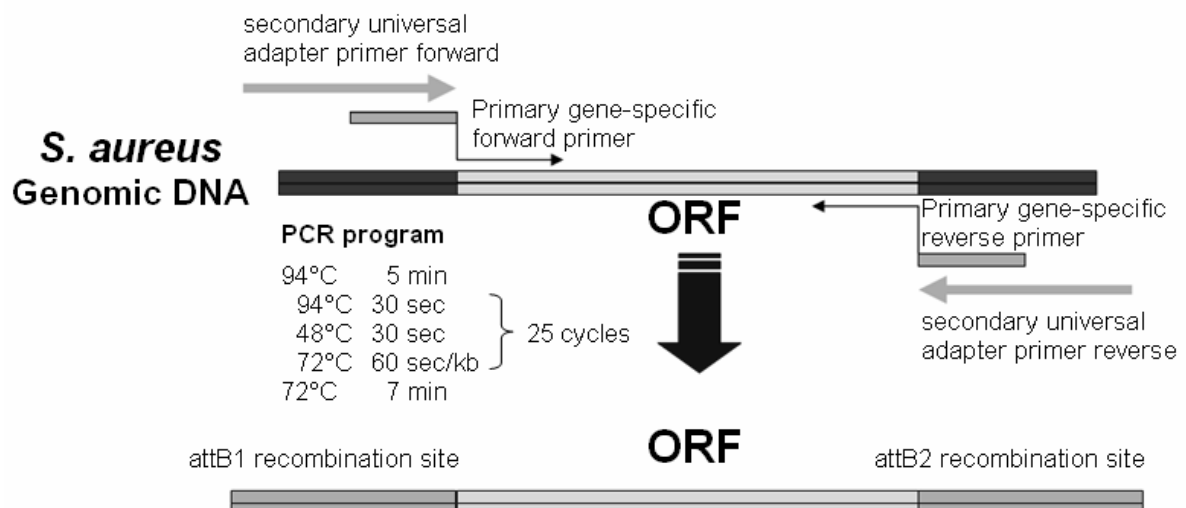

Supplement: Additional file 2 — Gateway recombination site attachement. This file shows the scheme of the one step adapter-PCR to create Gateway® adaptable attB PCR products. [file 1471-2164-9-321-S2.pdf]
